# Assessing a multicomponent intervention to improve quality of life in individuals with Long COVID (COVIDL/MIQoL): study protocol for a randomized controlled trial

**DOI:** 10.3389/fpubh.2025.1604971

**Published:** 2025-05-19

**Authors:** Mireia Morera, Antonio Arévalo, Cristina Garriga, Marta Corral-Magaña, Mari Carmen García-Arqué, Marta Gragea-Nocete, Cristina Pérez Díaz, Ramon Roca, Maria Llistosella

**Affiliations:** ^1^Primary Health Care, Consorci Sanitari de Terrassa, Barcelona, Spain; ^2^Fundació Joan Costa Roma, Consorci Sanitari de Terrassa, Barcelona, Spain

**Keywords:** Long COVID, randomized controlled trial, quality of life, multicomponent intervention, mental wellbeing

## Abstract

**Introduction:**

The impact of Long COVID on the quality of life of affected individuals is significant, therefore, the aim of this study is to analyse the effectiveness of the multicomponent intervention protocol to improve quality of life in individuals with Long COVID.

**Methods:**

A randomized controlled trial with two parallel arms will be conducted. Individuals diagnosed with Long COVID, but without any severe mental disorders, will be recruited. The sample size is estimated to be around 54 participants per group. A psychologist and a physiotherapist will carry out the intervention between January and March 2025. Participants will receive specific training in psycho-education and physical rehabilitation consisting of 18 sessions, to be held twice a week. Data collection will start in January 2025 and will finish in October 2025. Data will be collected: at baseline, before the intervention (T0); after 9 weeks, post-intervention (T1); and after 24 weeks, follow-up (T2), and will assess quality of live, well-being, anxiety, depression resilience, fatigue, and physical activity. An intention-to-treat analysis will be performed and the effect size will be calculated using Cohen’s d. All statistical analyses will be performed using R software version 4.2.2, with a 95% confidence level and a statistical significance level of *p* < 0.05.

**Discussion/conclusion:**

The results will be disseminated to individuals with Long COVID and their families throughout their primary health care center. Healthcare professionals will receive specific training to be able to develop and implement the intervention. In addition, the results will be disseminated to the scientific community via conferences and publications.

**Clinical trial registration:**

https://register.clinicaltrials.gov/prs/beta/studies/S000ENW100000080/recordSummary, Identifier NCT06492590.

## 1 Introduction

Coronavirus disease 2019 (COVID-19), caused by the severe acute respiratory syndrome 2 coronavirus (SARS-COV-2), can have persistent symptoms (1) and significant long-term health effects. The persistence of symptoms after acute SARS-CoV-2 infection, known as persistent COVID (COVID-P) or Long COVID (2), has emerged as a wide-ranging public health challenge. This condition, characterized by a wide range of physical and psychological symptoms that persist for at least 12 weeks following acute COVID-19 infection, exhibits symptoms that are not attributable to causes other than SARS-CoV-2 infection (3).

Many symptoms that could be classified as Long COVID have been described in the literature. The most common are: fatigue, fever, dry cough, dyspnoea, anosmia, ageusia, arthralgia, myalgia, diarrhea, headaches, cardiorespiratory problems, emotional disorders, mental fog, sleep disorders, cognitive disorders and mood disorders (3). A systematic review conducted by Nassarie et al. (1) identified more than 80 symptoms, highlighting that the most frequently were: shortness of breath or dyspnoea, fatigue or exhaustion, and sleep disorders or insomnia (1). The prevalence of depression and anxiety was estimated in 20% of cases, 2 years after COVID-19 (4). Additionally, one of the most prevalent symptoms was cognitive impairment, which leads to a significant degree of disability and poor quality of life (5).

In individuals who have experienced COVID-19, 10–20% may have long-term symptoms (6). However, this percentage could change according to the specific definition of the condition (7), as well as the severity of acute COVID-19 disease, which ranged from 6 to 46% in non-hospitalized individuals (7, 8). Approximately 200 million people worldwide live with long-term symptoms of COVID-19, according to estimates by a systematic review involving meta-analysis that was conducted by Chen et al. (8). More recently, another meta-analysis reported a prevalence of 30%, two-years after COVID-19 (4).

The impact of Long COVID on the quality of life of affected individuals is significant (9). Their quality of life is reduced, as is their ability to work. Physically, patients may experience chronic fatigue, pain, breathing difficulties, and a wide variety of the symptoms listed above, and this may limit their ability to carry out daily activities (9, 10).

Several recommendations have been made for the therapeutic management of Long COVID in primary care, with multidisciplinary rehabilitation, comprising physical, psychological and psychiatric aspects, being the treatment of choice (11). More specifically, according to NICE guidelines (11), multidisciplinary rehabilitation could include: providing information and education, supportive self-care, peer support, symptom management strategies and physical rehabilitation. Similarly, the Stanford Hall consensus proposes a multidisciplinary approach including: pulmonary/cardiac rehabilitation, exercise therapy, psychological management, musculoskeletal symptom management, neurorehabilitation, and medical counseling (12).

Given the multidimensional impact of Long COVID, a holistic approach to the disease and all its implications must be sought in order to improve the quality of life of affected individuals. To the best of our knowledge, in the primary care setting, there are few published clinical trials detailing the use of multicomponent interventions involving psycho-educational and physical rehabilitation activities to improve the quality of life of individuals with Long COVID. Among these, we can highlight the NUTESCOTI project: an intervention program including RVI that uses the MK Player 360 projector and includes mindfulness exercises, cognitive stimulation and physical rehabilitation (13); and an online physical and mental health rehabilitation program, called the REGAIN study (14). Positive outcomes, such as an increase in the quality of life of the participants (13) and improving cognition (14), were obtained from these projects.

Furthermore, the study conducted by Nikrah et al. (15) examined the efficacy of acceptance and commitment therapy on resilience and health-related quality of life in patients with post-acute COVID-19 syndrome. The results of this study showed a significant increase in resilience that was sustained at 3 months (15).

The World Health Organization has now called for priority to be given to the rehabilitation of the emotional, physical and cognitive consequences of COVID-19 (16). In this line, the present study aims to assess an innovative, multicomponent intervention, including psycho-education and physical rehabilitation, which is aligned with the complex needs of individuals with Long COVID.

## 2 Study objectives and hypotheses

### 2.1 Objectives

Assess the effectiveness of a multicomponent intervention in individuals with Long COVID.

Increase the quality of life, mental wellbeing, resilience, and physical condition in individuals with Long COVID.

Decrease the incidence of anxiety, depressed moods and fatigue in individuals with Long COVID.

### 2.2 Research hypothesis

We hypothesize that participants with Long COVID who receive the multicomponent intervention (psycho-education and physical rehabilitation) will experience an improvement in their quality of life, mental wellbeing, resilience, physical condition and cognitive functions, and a reduction in anxiety, depressed moods and fatigue, compared to the control group.

## 3 Methods and analysis

### 3.1 Design

This study will be a Randomized controlled trial with parallel arms. It will follow the recommendations of the Consolidated Standards of Reporting Trials (CONSORT) (17). The SPIRIT guidelines will also be followed (18). The trail has been registered in as Clinical trial NCT06492590.

### 3.2 Study setting and data collection

Individuals already diagnosed with Long COVID will be contacted by health professionals from different hospitals and centers belonging to the Consorci Sanitari de Terrassa (CST). Their eligibility will be assessed by these professionals and the principal researcher (MLL) in line with the established inclusion criteria. Two researchers (MLL, MCG) will then contact the candidates by telephone to offer them the opportunity to participate in the trial and they will be sent an online fact sheet with details of the study. Those who agree to participate must then sign the informed consent. Participants who give informed consent will be randomized into intervention and control groups. Once randomized, those in the intervention group will be subdivided into 4 subgroups of 13–14 participants. Each subgroup will receive the multicomponent intervention (psycho-education and physical rehabilitation).

Recruitment will take place between October and December 2024. A psychologist and a physiotherapist will carry out the intervention between January and March 2025. These professionals involved in the intervention will be trained during two training sessions about Long COVID and activities included in the intervention.

**Figure 1:**
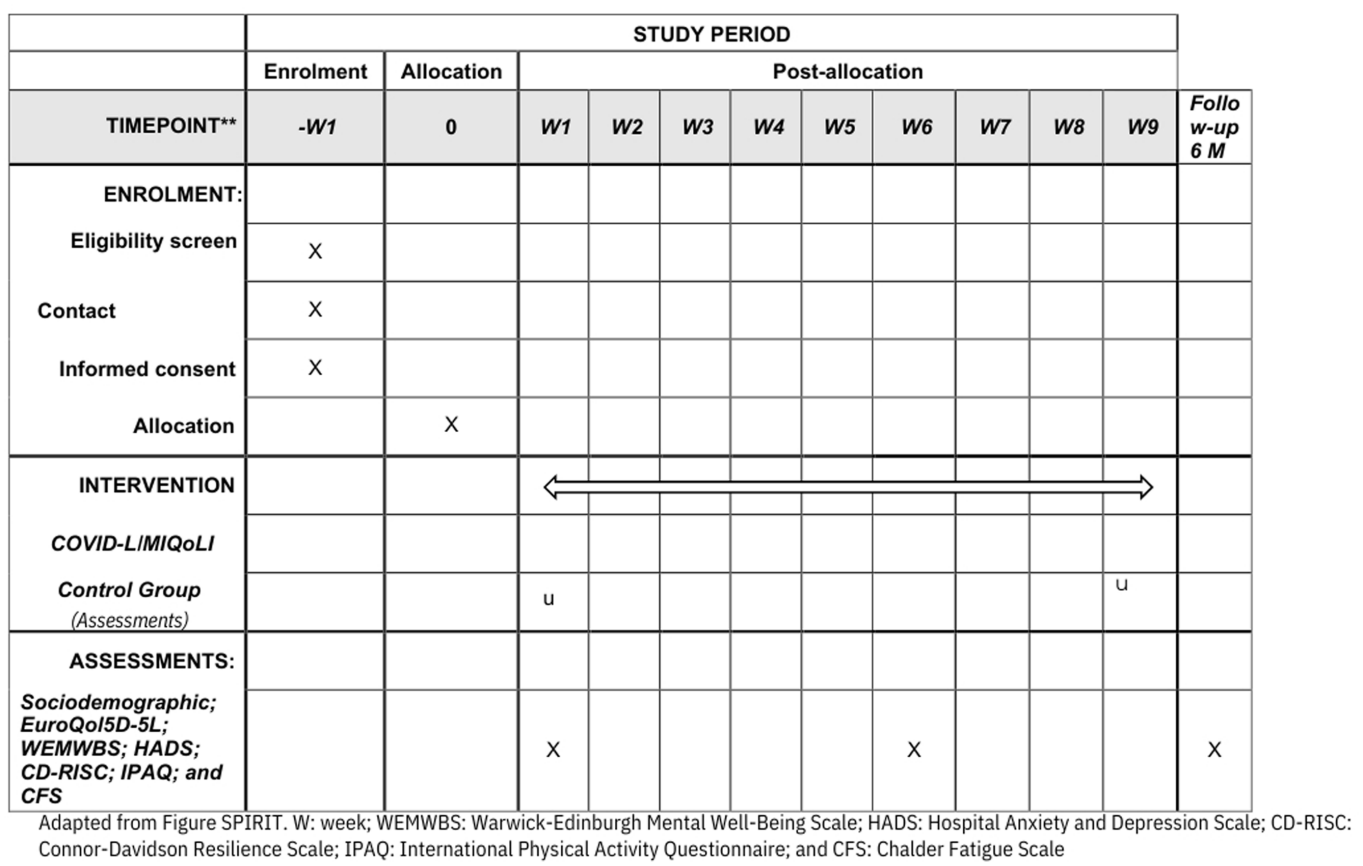
COVID-L/MIQoLI intervention: template enrolment, intervention, and assessments. WEMWBS, Warwick-Edinburgh Mental Well-Being Scale; HADS, Hospital Anxiety and Depression Scale; CD-RISC, Connor-Davidson Resilience Scale; IPAQ, International Physical Activity Questionnaire; CFS, Chalder Fatigue Scale.

**Figure 2:**
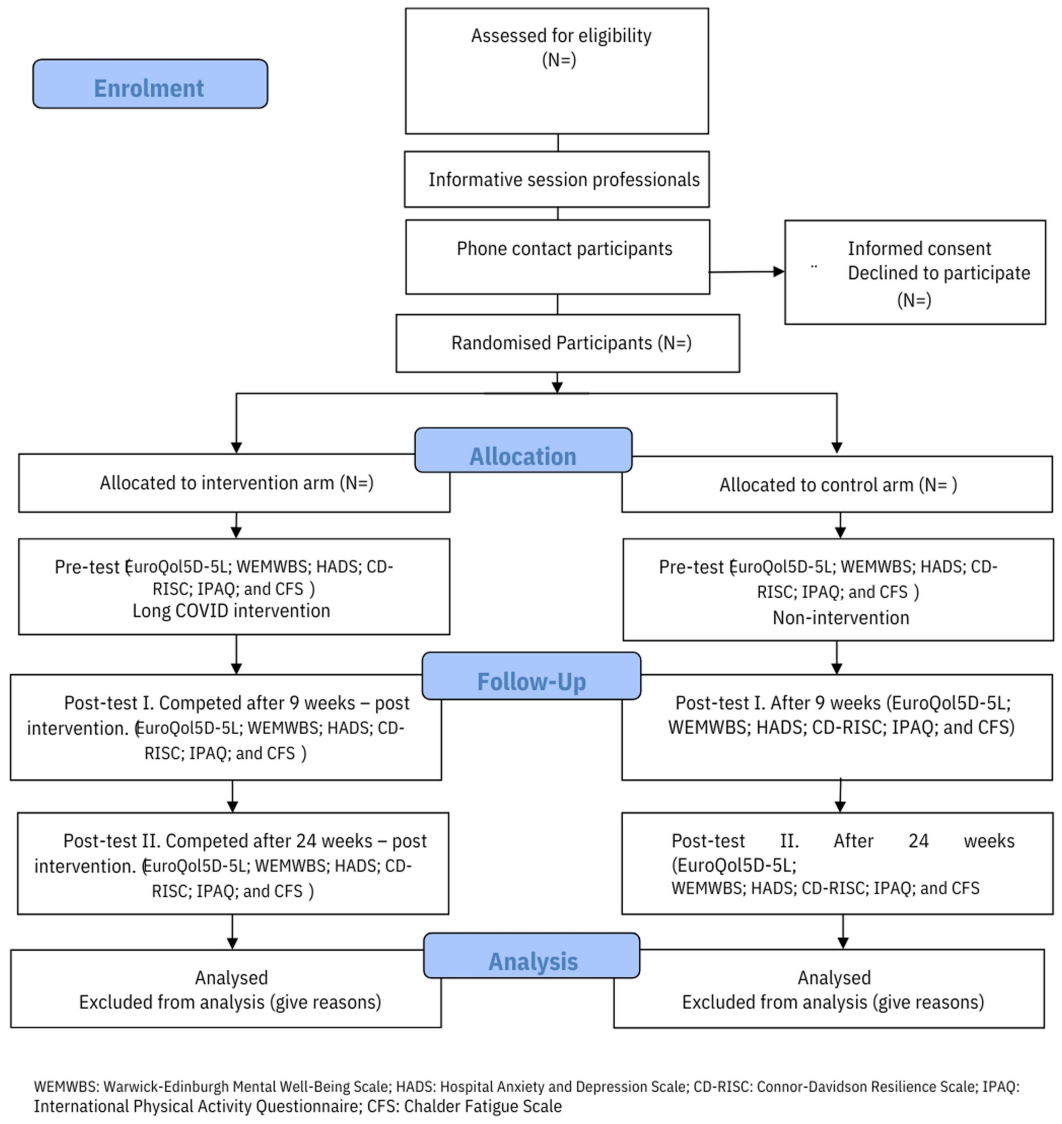
Consolidated Standards of Reporting Clinical Trial (CONSORT) flow chart. WEMWBS, Warwick-Edinburgh Mental Well-Being Scale; HADS, Hospita Anxiety and Depression Scale; CD-RISC, Connor-Davidson Resilience Scale; IPAQ, International Physical Activity Questionnaire; CFS, Chalder Fatigue Scale.

Data collection will start in January 2025 and end in September 2025. Data will be collected via a digital platform, at the baseline (T0), 9 weeks after the intervention (T1), and after 24 weeks of follow-up (T2) (see Figure 1). Participant data will be obtained using self-reported psychometric scales provided by the APP. Throughout the study, data will be stored on a secure server, in line with Spanish regulations. Recruitment of participants will be performed according to CONSORT guidelines for randomized clinical trials (see Figure 2) (17).

### 3.3 Sample size

The sample size has been estimated on the basis of the main quality of life variable and will be assessed using the EuroQol5D-5 L scale (19). Accepting an alpha risk of 0.05 and a statistical power greater than 0.8 in a bilateral contrast, 34 subjects in group 1 and 34 in group 2 are needed to detect a difference equal to or greater than 0.15 units. It is assumed that the common standard deviation is 0.21 (range between 0.20 and 0.25). A follow-up loss rate of 0.1 (10%) has been estimated. The GRANMO tool (20) was used to calculate the sample size.

### 3.4 Participants

#### 3.4.1 Inclusion criteria

Eligibility of individuals with a diagnosis of Long COVID made according to the WHO interim guideline (21).

Over 18 years of age and under 75 years of age.

Signing the informed consent form.

#### 3.4.2 Exclusion criteria

Severe sensory disabilities.

Physical illnesses that do not allow participation in the sessions.

Severe mental disorders that make it difficult to participate in groups. This includes schizophrenia spectrum disorders, bipolar disorder with psychotic features, major depressive disorder with psychotic symptoms, or any other psychiatric condition that, in the opinion of the researcher, significantly impairs the participant’s ability to participate in group activities safely and effectively.

Travel, surgery, or any other incident known in advance that does not allow the participant to complete at least 80% of the sessions.

Language difficulties.

### 3.5 Randomization and blinding

The participants will be randomly assigned to either the intervention or the control group by the external researcher (MC), using computer-generated random numbers. The participant groups and personnel (research team) will not be blinded given the difficulty of masking the conditions.

### 3.6 Intervention

#### 3.6.1 Intervention group

The multicomponent intervention was developed by an expert committee consisting of: psychologists, nurses, physiotherapists and people with Long COVID. It was developed following the framework of Central Sensitivity Syndrome, using empirical evidence and contextual information.

The psycho-education will consist of 9 sessions of 90 min: one per week, in groups with a maximum of 16 participants. The topics covered during these sessions with a specialized therapist will include: (1) education in neuroscience relating to the symptomatology of Long COVID; (2) Mindfulness; (3) Cognitive-Behavioral Therapy; and (4) Strategies for self-healing. The physical rehabilitation will consist of 9 sessions of 60 min: one per week, conducted by an expert physiotherapist. Each session will work on 4 areas, following a progressive approach: (1) warm-up and aerobic exercises (25 min); (2) functional and muscle-strengthening exercises (10 min); (3) proprioception exercises (10 min); and (4) breathing exercises (10 min). In order to address the variability of symptomatology and functional capacity as a function of age, we have planned to individually tailor physical activity interventions based on the baseline condition and needs of each participant. This personalized approach aims to ensure that all participants, regardless of age, can safely and effectively participate in the program (see Table 1).

**Table 1:** COVIDL/MIQoL outline of intervention sessions.

Session goals	Activities
*Session 1*: Introducing Long COVID and Central sensitization Knowing the group Explaining the study and the intervention Introduction to the main concepts on which the intervention was based Learning the concept of Long COVID and Central sensitization Introduction to conscious breathing	Presentations Describe the characteristics of the intervention and the key components on which it is based (1) Education in the neuroscience of Long COVID symptomatology; (2) Mindfulness; (3) Cognitive-Behavioral Therapy; (4) Self-care strategies Explain the concept of Long COVID and Central sensitization Deep breathing **Home practice**: Practice Deep Breathing every day
*Session 2*: Physical rehabilitation Reduction of sedentary behavior Increase physical activity between 5 and 10% each week	Individual prescription of physical activity Use the FITT model (F: Frequency, I: Intensity, T: Time, T: Type of activity) Each session will work on strength, aerobic endurance, balance and/or walking and flexibility activities, following general recommendations for the prescription of physical exercise **Home practice**: Exercises and physical activity
*Session 3*: The autonomous nervous system Understanding the functioning of the nervous system and its relationship to symptomatology Understanding the process of symptomatology maintenance in order to be able to reverse it Introduction to mindfulness	Basic concepts: the brain. Basic anatomy and functions; the autonomous nervous system. The Relationship to: Long COVID symptomatology, Emotions, and Polyvagal Theory Mindfulness: Introduction and Body Scan **Home practice:** Practice Body Scan 3 times a week. Make a daily record of activity (energy management planning)
*Session 4*: Physical rehabilitation	Individual prescription of physical activity **Home practice**: Exercises and physical activity
*Session 5*: Effective personal energy management Learning how to manage energy in order to improve the Long COVID symptoms Learning about food that contributes to recovery	Pacing: effective energy management: energy, availability and expenditure Types of fatigue. Classification of activities Strategies to reduce energy-draining activities Re-planning energy-enhancing activities Feeding: types of food that contribute to recovery. Types of food that hinder recovery **Home practice:** Identify and implement a feeding goal Re-planning the daily record of activity (energy management planning)
*Session 6*: Physical rehabilitation	Individual prescription of physical activity **Home practice**: Exercises and physical activity
*Session 7*: Emotional management Understanding how emotions function Understanding the relationship between emotions and symptomatology in Long COVID Learning to manage emotions	Concept of emotion; main emotions; functions of emotions; components of emotions. Emotional validation and management. How and why to validate emotions. Techniques to release unpleasant emotions Exercise: Emotional Mindfulness **Home practice:** Update and follow through with energy management planning. Identify a pleasant emotion each day
*Session 8*: Physical rehabilitation	Individual prescription of physical activity **Home practice**: Exercises and physical activity
*Session 9*: Cognitive management I Understanding the functioning of thought Understanding the relationship between thoughts and emotions and the symptomatology of Long COVID Learning to manage thoughts to contribute to well-being	Thoughts: Nature of thoughts; relationship with emotions, behavior and symptomatology in Long COVID; cognitive distortions, and cognitive restructuring Mindfulness: Through the storm **Home practice**: Select two recurrent cognitive distortions and generate useful alternative thought. Select two words related to the symptomatology and search for kinder terms
*Session 10*: Physical rehabilitation	Individual prescription of physical activity **Home practice**: Exercises and physical activity
*Session 11*: Cognitive management II Applying new, more useful ways of relating to thoughts Learning to manage recurring concerns	Cognitive defusion; thought distraction; and recurrent concerns Sleep habits **Home practice:** Identify and implement a sleep health goal and a new healthy eating goal
*Session 12*: Physical rehabilitation	Individual prescription of physical activity **Home practice**: Exercises and physical activity
*Session 13*: Acceptance of limits and self-compassion Learning to accept one’s limits Practicing self-compassion	Acceptance of personal limits: What is it and why is it necessary? Acceptance exercises Self-compassion: What is it and why is it necessary? Mindfulness: self-compassion exercise **Home practice:** Identify and implement a new sleep health goal
*Session 14*: Physical rehabilitation	Individual prescription of physical activity **Home practice**: Exercises and physical activity
*Session 15*: Assertiveness, limits and gratitude Learning to communicate assertively Learning to set healthy limits in relationships with others Learning to practice gratitude	Assertiveness: What is it and why is it necessary? Communication styles. Exercise: how to explain what happens to people and what they need Setting limits: How and why. Exercise: Saying “NO” Gratitude: Benefits and Gratitude journal **Home practice:** Identifying and implementing a new healthy eating goal. Start a gratitude journal
*Session 16*: Physical rehabilitation	Individual prescription of physical activity **Home practice**: Exercises and physical activity
Session 17: Review of content Review and integration of knowledge acquired	Review of the contents of all the sessions: contents, news tools, and doubts Self-assessment exercise Feedback from participants
*Session 18*: Physical rehabilitation	Individual prescription of physical activity

To ensure fidelity to the intervention and adherence of the participants, a series of exercises to be done at home after each session is contemplated. In addition, reminders will be sent to participants about upcoming sessions to encourage them to attend, and attendance will be registered for each of the sessions (see Table 1).

#### 3.6.2 Control group

Participants in the control group will complete the same research questionnaires as those in the intervention group and during the same period (January to September 2025). Participants in the control group will be placed on a waiting list to receive the intervention once its effectiveness has been demonstrated.

### 3.7 Monitoring procedure and risk participants

This clinical trial does not test participants for medications or perform any invasive tests. It can therefore be considered low risk and no specific insurance will be taken out. However, the research team agrees that in the case of detecting a participant with a clear risk, such as depression, or with a re-exacerbation of Long COVID symptomatology, such as dyspnoea, the therapist, physiotherapist and research team must inform the participant’s referring doctor/nurse at their primary health care center. If the participant suffers a crisis or an exacerbation that requires urgent attention, either during the intervention sessions or the study, the emergency service of the primary care center where the sessions are taking place will be notified in order to immediately assess the participant’s condition. A member of the participant’s family will also be contacted if necessary, and/or if requested by the participant. Confidentiality will be maintained at all times to avoid the stigmatization of the participant.

### 3.8 Outcomes measures

#### 3.8.1 Primary outcomes

Quality of life will be the primary outcome and will be measured using the EuroQol5D-5L scale (19). A review of the literature concluded that this scale has excellent psychometric properties and covers a wide range of populations, conditions and contexts (22). It has also been used in several studies of psycho-educational and physical rehabilitation interventions in individuals with Long COVID (9, 14).

The scale analyses five health dimensions, with one item per dimension: mobility (MO), self-care (SC), usual activities (UA), pain/discomfort (PD) and anxiety/depression (AD). Each item has three levels of severity: no problems, some or moderate problems, and severe problems (19). The second part of the EQ-5D consists of a 20-centimeter, millimeter vertical Visual Analog Scale (VAS) ranging from 0 (the worst imaginable health) to 100 (the best imaginable health) (19).

#### 3.8.2 Secondary outcomes

Well-being will be assessed using the Warwick-Edinburgh Mental Well-Being Scale (WEMWBS); this is a 5-point Likert-type scale comprising 14 items. The total score ranges from 14 to 70 points. Higher scores indicate higher levels of mental well-being. The Spanish validation showed that, on the original scale, a confirmatory factor analysis adjusted to a one-factor model (CFI = 0.974; TLI = 0.970; RMSEA = 0.059; v2 = 584.82; df = 77; *p* = 0.001). This scale also has a high degree of internal consistency (Cronbach’s alpha = 0.930; Guttman’s lambda-2 = 0.932) (23).

Mental health problems, such as anxiety and depression, will be assessed using the Hospital Anxiety and Depression Scale (HADS) (24). This is a 4-point Likert-type scale (0–3) with a total score ranging from 0 to 21 for each subscale, in which higher scores are indicative of greater symptom severity. The Spanish validation reported a Cronbach’s alpha of 0.90 for the whole scale, 0.84 for the Depression subscale, and 0.85 for the Anxiety subscale (24).

Resilience will be measured using the Connor-Davidson Resilience Scale (CD-RISC) (25, 26). This is a self-administered scale consisting of 10 Likert-type items with five response options (0 = not true, 1 = rarely true, 2 = sometimes true, 3 = often true, and 4 = true most of the time). The scale measures the ability to cope with stress and is designed for use with clinical samples. The scale score correlations range from 0.45 to 0.69, and Cronbach’s alpha was 0.85 (25). Higher scores indicate higher levels of resilience (25).

Physical condition will be assessed using the International Physical Activity Questionnaire (IPAQ) (27) and the Chalder Fatigue Scale (28). The short-self-administered version of the IPAQ consists of five questions about the frequency, duration and intensity (vigorous and moderate) of physical activity in the last 7 days, as well as the time spent walking and sitting on weekdays. The specificity of the questionnaire for detecting non-compliance with physical activity recommendations is 75%. The sensitivity is 75%. The Kappa coefficient was low (= 0.33, *p* < 0.05) (27).

The Spanish version of the Chalder Fatigue Scale consists of 14 items classified into 4 dimensions (low energy, sound problems, concentration problems, and subjective cognitive dysfunction). Respondents rated each item according to their experience over the previous month, using a 4-point Likert scale. The total scores range from 0 to 42. Higher scores indicate greater severity of fatigue (28).

Finally, we will consider socio-demographic variables selected from the recommendations for harmonization of research in adults with COVID-19, established by 107 experts from 19 countries (29). The following variables will be included: age, gender, employment status, civil status, nucleus of conviviality, associated chronic pathology; COVID-19 vaccines, socio-economic data, country, and Long COVID symptoms.

The data will be managed by a statistician external to the research team and stored in a secure storage system belonging to the Consorci Sanitari de Terrassa.

### 3.9 Data analysis

A descriptive analysis of the variables will be carried out, analysing the frequencies and percentages of the qualitative variables; the mean and standard deviations for the quantitative normal distributed variables and the median; and the quartiles for the quantitative non-normally distributed variables. An intention-to-treat analysis will be performed. The values of the scores for the intervention and control groups will be compared using the t-student test, if the score is normally distributed; using the Mann–Whitney test, if the score is not-normally distributed; and using the chi-square test, if the score has a cut-off. The effect size will be assessed using Cohen’s d, with its corresponding confidence intervals.

A linear regression and a logistic regression will be considered, including any other variables that could potentially affect the response. We will also stratify the results for factors, such as gender, age and symptomatology, while others may also be considered. The Kolmogorov–Smirnov and Shapiro–Wilk tests will be used to assess normality. All the confidence intervals will be performed with a 95% confidence level. All analyses will be carried out using R software version 4.2.

## 4 Discussion

At present, there are still not enough interventions to improve the quality of life of individuals with Long COVID. In this protocol, we present a new, comprehensive, multicomponent intervention which can be developed and implemented at primary health care centers.

This study describes the design and implementation of an approach for evaluating the effectiveness of this innovative intervention.

One of the limitations of this study is that the long-term effects of the intervention may be lost over time. We propose making an evaluation after 6 months in order to assess the duration of the effect and to see whether it is necessary to either reinforce the intervention, or to repeat it after 6 or 7 months, in order to maintain the results.

Once the effectiveness of this intervention has been evaluated, the materials used during the sessions will be made available for replication at interested primary care centers.

In summary, given the multidimensional impact of Long COVID and the limitations of current approaches, this project seeks to offer an innovative intervention that is aligned with the, often complex, needs of patients. There may, however, be some variation in the effectiveness of this intervention between groups due to differences between the teams involved. If this should be the case, we propose improving training in order to ensure that teams act as similarly as possible.

We expect that this intervention will offer a significant improvement in the quality of life and mental well-being of individuals with Long COVID and mark a significant step forward in the treatment of Long COVID.

We are currently finishing recruitment at www.clinicaltrials.gov. Any possible modifications to the study will be monitored by the innovation and research department of the Consorci Sanitari de Terrassa. These modifications will then be updated with the Quality Evaluation Agency of Catalonia and in the protocol registry for clinical trials. Any changes will be included in an article making a final assessment of the intervention.

## 5 Ethics and dissemination

The present protocol was reviewed and approved by the Ethics Committee Institutional Review Board of the “Consorci Sanitari de Terrassa” (Ref: 01-24-1CR-068), in June 2024. The study will be carried out in compliance with current legislation, following the Biomedical Research Law 14/2007. The confidentiality of participant data will be guaranteed in compliance with Organic Law 3/2018, of 5 December, on the Protection of Personal Data, and with the guarantee concerning digital rights and the General Data Protection Regulation (EU) 679/2016. This intervention should not cause any physical or psychological harm to participants. However, a risk monitoring protocol has been developed, as explained above.

The results will be shared with individuals with Long Covid and their families through their primary healthcare center, while the research team will inform the local media. Additionally, healthcare professionals will receive specialized training to develop and implement the intervention in hospitals and primary care centers. Finally, we will disseminate our findings to the scientific community via conferences and publications.
